# Effect of structured nursing care on treatment tolerance and quality of life in patients with esophageal cancer undergoing radiotherapy: a retrospective cohort study

**DOI:** 10.3389/fonc.2026.1811695

**Published:** 2026-09-23

**Authors:** Chengyu Zhao, Xuefeng Huang, Yingying Han, Jia Fu, Hongli Zhang

**Affiliations:** 1 Department of Radiation Therapy, Thoracic Radiotherapy Ward III, Shanxi Provincial Cancer Hospital/Shanxi Hospital, Cancer Hospital, Chinese Academy of Medical Sciences/Third People's Hospital Affiliated to Shanxi Medical University, Taiyuan, Shanxi, China; 2 Department of Radiation Oncology, Abdominal Radiotherapy Ward, Shanxi Provincial Cancer Hospital/Shanxi Hospital, Cancer Hospital, Chinese Academy of Medical Sciences/Third People's Hospital Affiliated to Shanxi Medical University, Taiyuan, Shanxi, China; 3 Endoscopy Center, Shanxi Province Cancer Hospital/Shanxi Hospital Affiliated to Cancer Hospital, Chinese Academy of Medical Sciences/Cancer Hospital Affiliated to Shanxi Medical University, Taiyuan, China; 4 Department of Radiation Oncology, Thoracic Radiotherapy Ward I, Shanxi Hospital, Cancer Hospital, Chinese Academy of Medical Sciences/Third People's Hospital Affiliated to Shanxi Medical University, Taiyuan, Shanxi, China

**Keywords:** esophageal cancer, nursing care, quality of life, radiation therapy, radiation-related toxicity, treatment tolerance

## Abstract

**Objective:**

To evaluate the relationship between structured nursing care (SNC) and treatment tolerance in patients with esophageal cancer receiving radiotherapy (RT).

**Methods:**

This single-center retrospective cohort analysis included 120 patients with esophageal cancer who received definitive or adjuvant RT from January 2020 to December 2024. Patients received either standard nursing care (standard care) or SNC. The primary outcome was RT completion. Secondary outcomes included grade ≥2 toxicity, unplanned hospitalizations, weight change, and quality of life (QoL) as measured by the EORTC QLQ-C30 and QLQ-OES18. Multivariable logistic regression was performed to adjust for prespecified covariates.

**Results:**

The SNC group had a higher percentage of patients completing RT compared with the standard care group (89.5% vs 79.4%, p = 0.14). The incidence of grade ≥2 toxicity was lower in the SNC group (33.3% vs 50.8%, p = 0.048). Unplanned hospitalizations were also lower in the SNC group (7.0% vs 17.5%, p = 0.08). Weight loss was less pronounced in the SNC group (−1.7 ± 1.9 kg vs −2.8 ± 1.9 kg, p = 0.02). Descriptive QoL change data showed directionally more favorable patterns in the SNC group compared with the standard care group. In the adjusted analyses, SNC was associated with a higher odds of RT completion, but that association did not reach statistical significance (OR 2.09, 95% CI 0.69–6.30; p = 0.1899).

**Conclusion:**

SNC was associated with directional trends favoring treatment tolerance outcomes, although the adjusted association with completion of RT was not statistically significant. Therefore, these findings are exploratory in nature and warrant additional prospective studies evaluating structured supportive models of care.

## 1 Introduction

Esophageal cancer is among the most prevalent malignancies and the leading cause of cancer-related death globally (1). Although multimodal treatments such as surgery, chemotherapy, and radiation therapy have improved patient outcomes, newly diagnosed esophageal cancer patients often present with locally advanced disease and require definitive or adjuvant radiation as part of their treatment plan (2). While radiation therapy helps control tumor growth and relieve symptoms, the treatment is associated with various adverse effects, including but not limited to esophagitis, dysphagia, fatigue, nutritional issues, and psychosocial distress; any of which could negatively affect the patient’s ability to adhere to treatment or lead to decreased quality of life overall (3–5).

Determining whether treatment was completed has become a significant measure of the quality of care and tolerance of treatment among radiotherapy patients (6). The interruption or early termination of radiation therapy has been associated with poor oncological outcomes such as lower survival rates and a higher chance of recurrence (7, 8). Thus, developing and improving supportive care strategies to reduce treatment-related toxicity and continue treatment is an important but under-addressed area of comprehensive cancer care.

Nursing care supports oncology through the performance of various activities; these activities include monitoring symptoms, providing nutrition support, educating patients, providing psychological support, and coordinating with multiple disciplines in caring for patients (9). Studies have shown that implementing structured nursing interventions improves symptom control and patient-reported outcomes in numerous malignancies receiving radiotherapy (10–12). However, there is limited available research establishing how structured nursing care for patients with esophageal cancer who receive radiotherapy impacts the completion of treatment, decreases treatment-related toxicity, hospitalizations, and improves quality of life in real-world settings.

The patient population with esophageal cancer, due to the difficulty in swallowing, creates a risk for treatment complications related to tumor-related dysphagia, initial nutritional deficits, and the additive effects of chemotherapy and radiotherapy (13). Therefore, there is a need for focused supportive care interventions to preserve functional status, ensure adequate nutrition, and maintain uninterrupted treatment. Despite clinical rationale for implementing structured nursing interventions, there is a lack of solid real-world evidence for this patient population.

From a translational standpoint, implementing structured nursing care may help connect the technical delivery of radiation therapy with patient-centered supportive care through systematic symptom assessment, early intervention, and coordinated multidisciplinary care (14, 15). Recent studies have also shown that structured supportive care models and symptom-management devices are important for improving patient outcomes during cancer therapy, thereby validating the value of this model of care in today’s oncology practice (11). Recent randomized evidence further supports this approach, demonstrating that enhanced supportive care frameworks during radiotherapy can improve patient-reported quality of life outcomes (16).

The aim of this retrospective cohort study was to evaluate whether receiving structured nursing care was associated with completion of radiotherapy, radiotherapy-related toxicity, hospitalization, weight change, and quality-of-life outcomes among patients with esophageal cancer receiving radiotherapy in a real-world setting.

The purpose of this study was to provide exploratory evidence regarding the association between structured nursing care and treatment-related outcomes.

## 2 Materials and methods

### 2.1 Study design and ethics approval

This study employed a retrospective observational cohort design at a tertiary academic institution specializing in oncology. Using electronic medical records, we identified consecutive adult patients diagnosed with histologically confirmed esophageal cancer who received definitive or adjuvant radiotherapy at the institution between January 2020 and December 2024. Eligible patients for inclusion in this study were those aged 18 or older with a complete baseline clinical assessment and complete radiotherapy planning data. Patients were excluded from the study for any of the following reasons: the presence of metastatic disease; palliative radiotherapy only (no definitive or adjuvant); a history of prior thoracic irradiation; or lack of complete treatment or follow-up data. A total of 120 eligible patients were included in the final analysis, comprising 63 patients in the standard nursing group and 57 patients in the structured nursing group. Baseline was defined as the date of radiation therapy initiation, from which all clinical outcomes and follow-up assessments were measured.

As this study was a retrospective analysis of routinely collected clinical data, no *a priori* sample size calculation was performed; all eligible patients during the specified study period were included to minimize selection bias. This study was conducted in accordance with STROBE guidelines for observational studies (17).

This study was approved by the Institutional Review Board of Shanxi Province Cancer Hospital (Approval No: KY2023088) and conducted in accordance with the Declaration of Helsinki. Given the retrospective design and the use of anonymized, routinely collected clinical data, the Ethics Committee waived the requirement for written informed consent, and patient confidentiality was strictly maintained throughout data collection and analysis.

### 2.2 Radiation therapy

All patients underwent computed tomography-based simulation and received intensity-modulated radiation therapy in accordance with institutional protocols and national guidelines. Target volumes were delineated, and the dose was prescribed by experienced radiation oncologists. The prescribed dose was typically between 50.4Gy and 60Gy delivered in 25–30 fractions. Chemotherapy was given concurrently with radiation therapy when it was clinically indicated, with consideration of the stage and performance status of the tumor.

A radiotherapy interruption was defined as ≥3 consecutive days of unintended lost treatment time (excluding weekends or scheduled breaks in the treatment program), as this was chosen as a relevant interruption to treatment due to treatment intolerance rather than routine scheduling variations. Also, cumulative days of interruption during radiotherapy were documented for descriptive purposes. Adequate treatment tolerance was evidenced by the completion of all scheduled radiotherapy without interruption or cessation.

### 2.3 Care models for treatment

The nursing care delivered to patients in this study consisted of two care models used throughout their enrollment.

Standard nursing care provided patients with routine nursing services after undergoing treatment for cancer. Nursing care routinely included vital sign monitoring, medication administration, basic dietary information, and routine discharge teaching.

Dedicated oncology nursing personnel provided structured nursing care using a coordinated supportive-care workflow implemented over the course of the radiotherapy treatment. This care model included scheduled weekly evaluations of treatment-related toxicities, systematic assessment of changing body weight, individualized nutritional counseling, and education on dietary and swallowing strategies. Patients were given educational support on identifying symptoms, adhering to treatment, and managing symptoms during the radiotherapy treatment, and were scheduled for psychological support in relation to treatment-related distress.

The care model incorporated predefined escalation pathways: patients who lost a significant amount of weight, who experienced dysphagia (difficulty swallowing), or were unable to consume sufficient calories through their diet were referred for nutritional interventions, including oral supplements or enteral nutrition, when clinically indicated, and those whose symptom burden worsened were referred back to the radiation oncology physician or rehabilitation services. There was routine multidisciplinary coordination of care among the oncology nurse, radiation oncologist, nutritionist, and rehabilitation staff to provide structured nursing interventions, all of which were documented in the clinical record.

Patients were assigned either to the structured nursing care or the standard oncology nursing care model based on the timing of the implementation of the program and the availability of structured nursing resources, rather than by a random assignment or patient preference; therefore, the comparisons between the two groups should be interpreted as observational and are subject to confounding variables.

### 2.4 Measures of outcomes

The primary measure of treatment tolerance was successful completion of planned radiation therapy without interruption or early termination.

Secondary outcomes included: incidence of increased severity (grade ≥2) of radiation therapy-related toxicity; number of unplanned hospitalizations that occurred during radiotherapy; change in body weight from the start of the radiation therapy through to completion of the radiation therapy; and patient perceived quality of life. The Common Terminology Criteria for Adverse Events (CTCAE) V5 (18) were used to assign scores to treatment-related adverse events.

Patients’ quality of life was evaluated using the EORTC Quality of Life Questionnaire Core (QLQ-C30) and the QLQ-OES18 (esophageal cancer-specific module), both validated instruments widely used in oncology research (19, 20).

Unplanned hospital admissions are those that occur during radiation therapy.

Weight change was calculated from the difference between the patient’s initial weight before starting radiation therapy and their final weight after completing the radiation treatment course.

Baseline clinical/demographic characteristics included age, sex, tumor stage, histologic subclassification, concurrent chemotherapy, baseline body mass index, and comorbidity status.

Performance status was not included in this analysis due to the incomplete availability across the retrospective dataset.

### 2.5 Statistical analyses

Continuous variables were summarized using either mean ± standard deviation or median with interquartile range, depending on their distribution; normality of the variables was assessed prior to statistical analyses. Between-group comparisons were made using independent-samples t-tests for normally distributed variables, and Mann-Whitney U tests for non-normally distributed variables. All categorical variables were compared using chi-square or Fisher’s exact tests, as appropriate.

Associations between the nursing care model and radiotherapy completion were assessed using multivariable logistic regression, including clinically relevant covariates (age, body mass index, tumor stage, and concurrent chemotherapy).

Adjusted odds ratios (ORs) with 95% confidence intervals (CIs) were reported. Given the non-randomized design and modest sample size, the multivariable analysis was considered exploratory and intended to evaluate the robustness of associations rather than establish causality.

Data on quality of life were evaluated descriptively, and differences from baseline to endpoint were reported as mean ± SD. Data were lacking to enable any formal longitudinal statistical comparisons.

A two-sided p-value < 0.05 was an indicator of statistical significance. All data were analyzed using SPSS (IBM Corp., Armonk, NY).

### 2.6 Missing data handling

Cases missing the primary outcome were excluded from the analysis.

For secondary outcomes, a complete-case analysis (when missingness was <5%) was performed, and quality-of-life analyses were conducted only among patients with baseline and post-treatment data available.

## 3 Results

### 3.1 Patient selection & baseline characteristics

Eligibility assessment was performed on 146 patients. After excluding 26 patients due to incomplete clinical records or prior radiotherapy, 120 patients were included in the final analysis. Sixty-three patients received a standard nursing assessment, and fifty-seven patients received a structured nursing assessment. The process of screening patients for eligibility and assigning them to treatment groups is shown in **Figure 1**.

**Figure 1:**
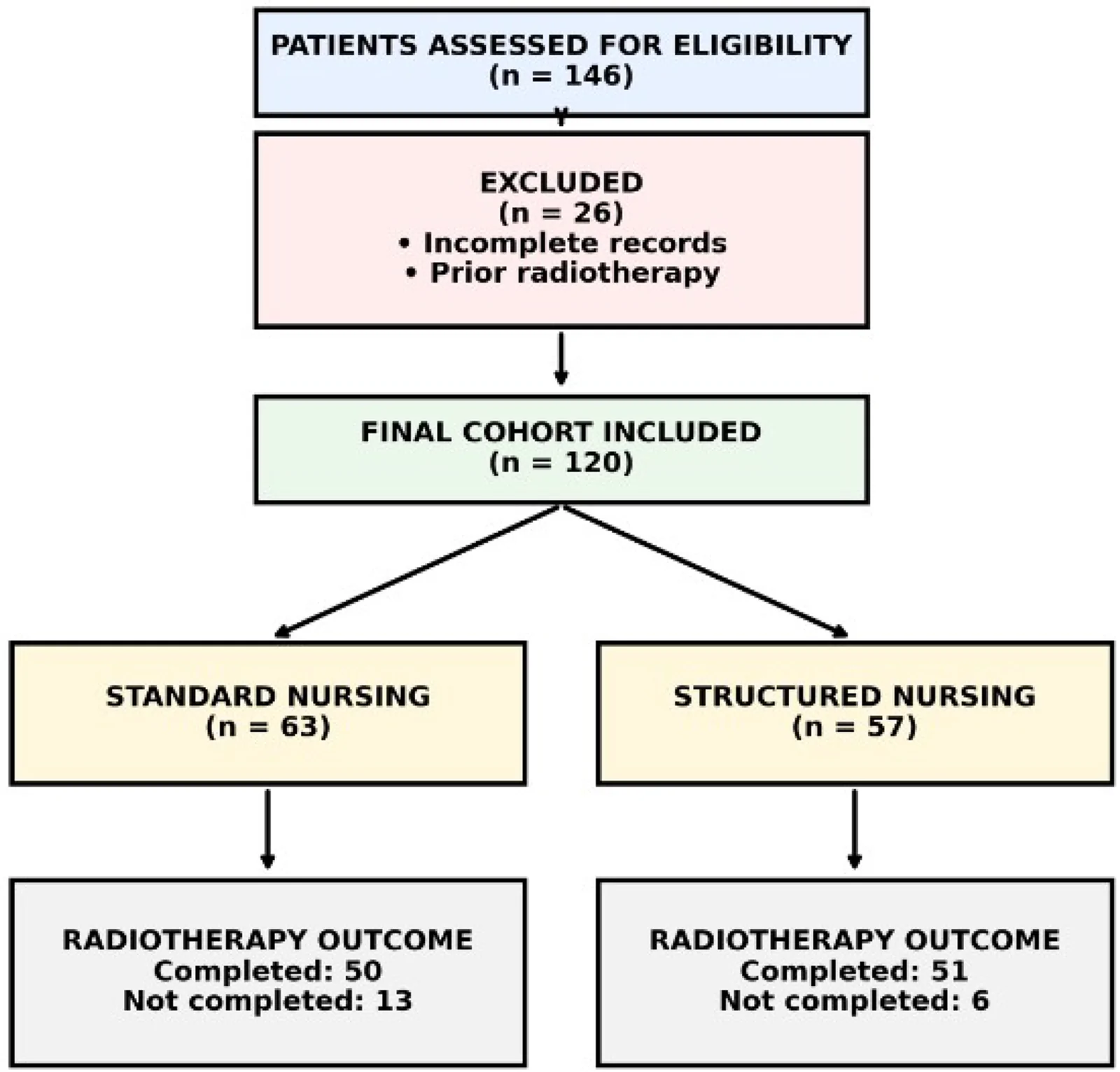
Flowchart of patient selection and study inclusion. A total of 146 patients were assessed for eligibility. After exclusion of 26 patients due to incomplete clinical records or prior radiotherapy, 120 patients were included in the final cohort. Patients were allocated to standard nursing (n = 63) or structured nursing (n = 57). The primary outcome, radiotherapy completion, is presented for both groups.

Baseline demographic and clinical characteristics of study participants are outlined in **Table 1**. The mean age of participants in structured nursing care and standard nursing care was comparable (60.9 ± 9.8 vs 61.4 ± 9.7 years, p = 0.78).

**Table 1:** Baseline characteristics of patients.

Characteristic	Structured nursing	Standard nursing	P-value
Age (years)	60.9 ± 9.8	61.4 ± 9.7	0.78
BMI (kg/m²)	23.3 ± 3.3	25.1 ± 3.4	0.06
Male, n (%)	29 (50.9%)	31 (49.2%)	0.85
Concurrent chemotherapy, n (%)	26 (45.6%)	27 (42.9%)	0.76
Stage ≥3, n (%)	45 (78.9%)	44 (69.8%)	0.24

Values are presented as mean ± standard deviation or number (%), as appropriate. Between-group comparisons were performed using Student’s t-test for continuous variables and chi-square or Fisher’s exact test for categorical variables. Stage ≥3 indicates locally advanced disease.

The two groups did not differ statistically in sex distribution (p = 0.85), BMI (p = 0.06), tumor stage (p = 0.24), or simultaneous chemotherapy (p = 0.76), suggesting comparable baseline characteristics.

### 3.2 Treatment tolerance & radiotherapy completion

A total of 89.5% of patients who received structured nursing care completed their planned course of radiotherapy, compared with 79.4% of patients who received standard nursing care (**Table 2**; **Figure 2**).

**Table 2:** Treatment tolerance and clinical outcomes.

Outcome	Structured nursing	Standard nursing	P-value
Radiation completion, n (%)	51 (89.5%)	50 (79.4%)	0.14
Grade ≥2 toxicity, n (%)	19 (33.3%)	32 (50.8%)	0.048
Hospitalization during RT, n (%)	4 (7.0%)	11 (17.5%)	0.08
Interruption days, median (IQR)	0 (0–0)	0 (0–0)	1.00
Weight change (kg), mean ± SD	-1.7 ± 1.9	-2.8 ± 1.9	0.02
Weight change (kg), median (IQR)	-1.5 (−2.8 to −0.6)	-2.6 (−3.9 to −1.4)	0.03

Radiation completion was defined as receipt of the planned radiotherapy course without premature discontinuation. Grade ≥2 toxicity was assessed according to CTCAE version 5.0. Hospitalization refers to any unplanned inpatient admission during radiotherapy. Interruption days represent cumulative unplanned treatment gaps. Continuous variables are presented as mean ± standard deviation or median (interquartile range), as appropriate.

**Figure 2:**
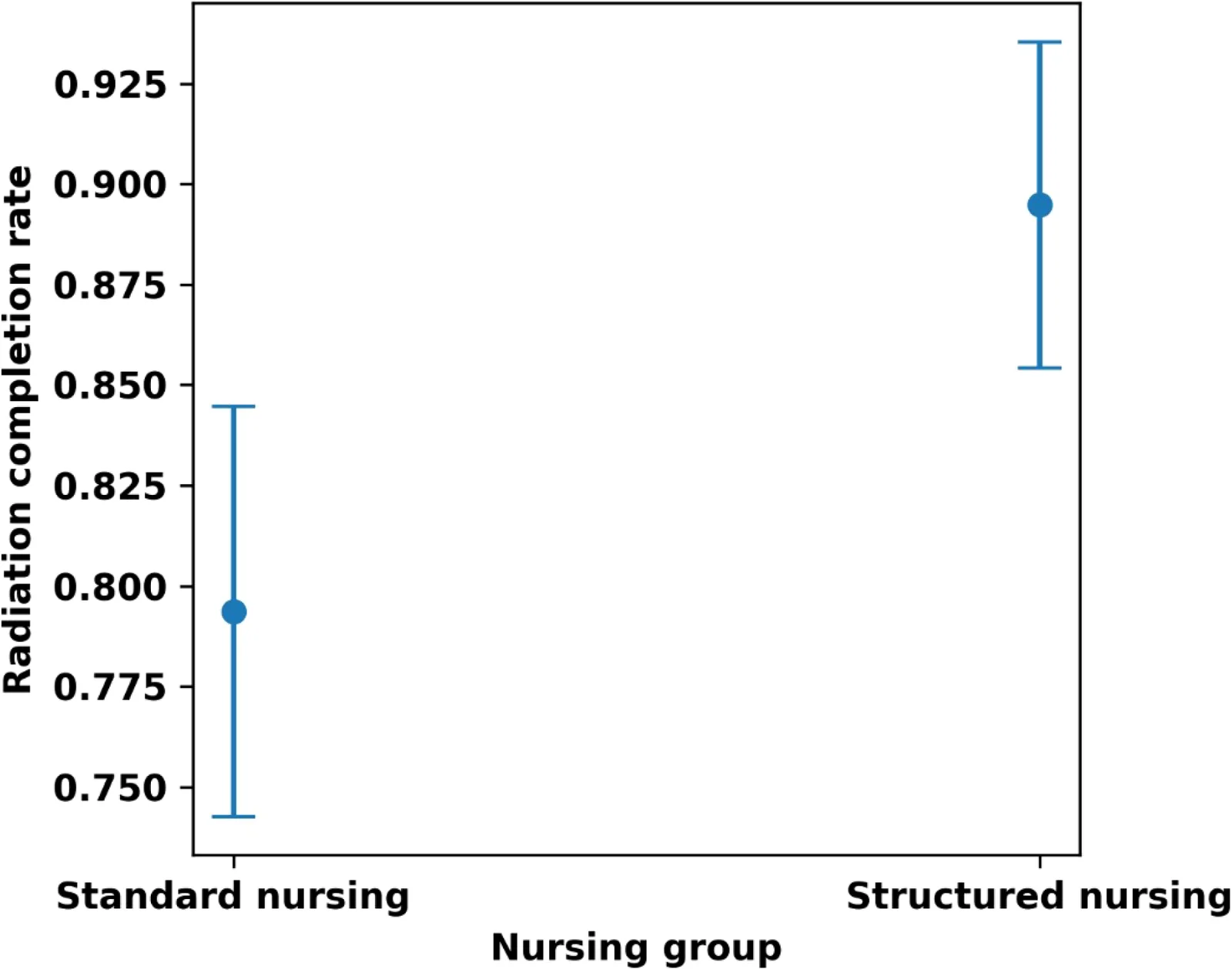
Radiotherapy completion rate according to the nursing care model. Comparison of radiotherapy completion rates between structured nursing and standard nursing groups.

While structured nursing had a higher completion rate for radiotherapy than standard nursing (p = 0.14), the difference was not statistically significant. The incidence of radiation-related toxicities of grade 2 or higher was lower in the structured nursing group (33.3%) than in the standard group (50.8%) (p = 0.048) (**Table 2**; **Figure 3**). Unplanned hospitalization during radiotherapy was lower in the structured nursing group (7.0%) than in the standard nursing group (17.5%), although this difference did not reach statistical significance (p = 0.08). Patients receiving structured nursing care experienced less weight loss (−1.7 ± 1.9 kg vs −2.8 ± 1.9 kg; p = 0.02) (**Figure 4**). When expressed as median (IQR), weight loss remained lower in the structured nursing group (−1.5 [−2.8 to −0.6] kg vs −2.6 [−3.9 to −1.4] kg; p = 0.03).

**Figure 3:**
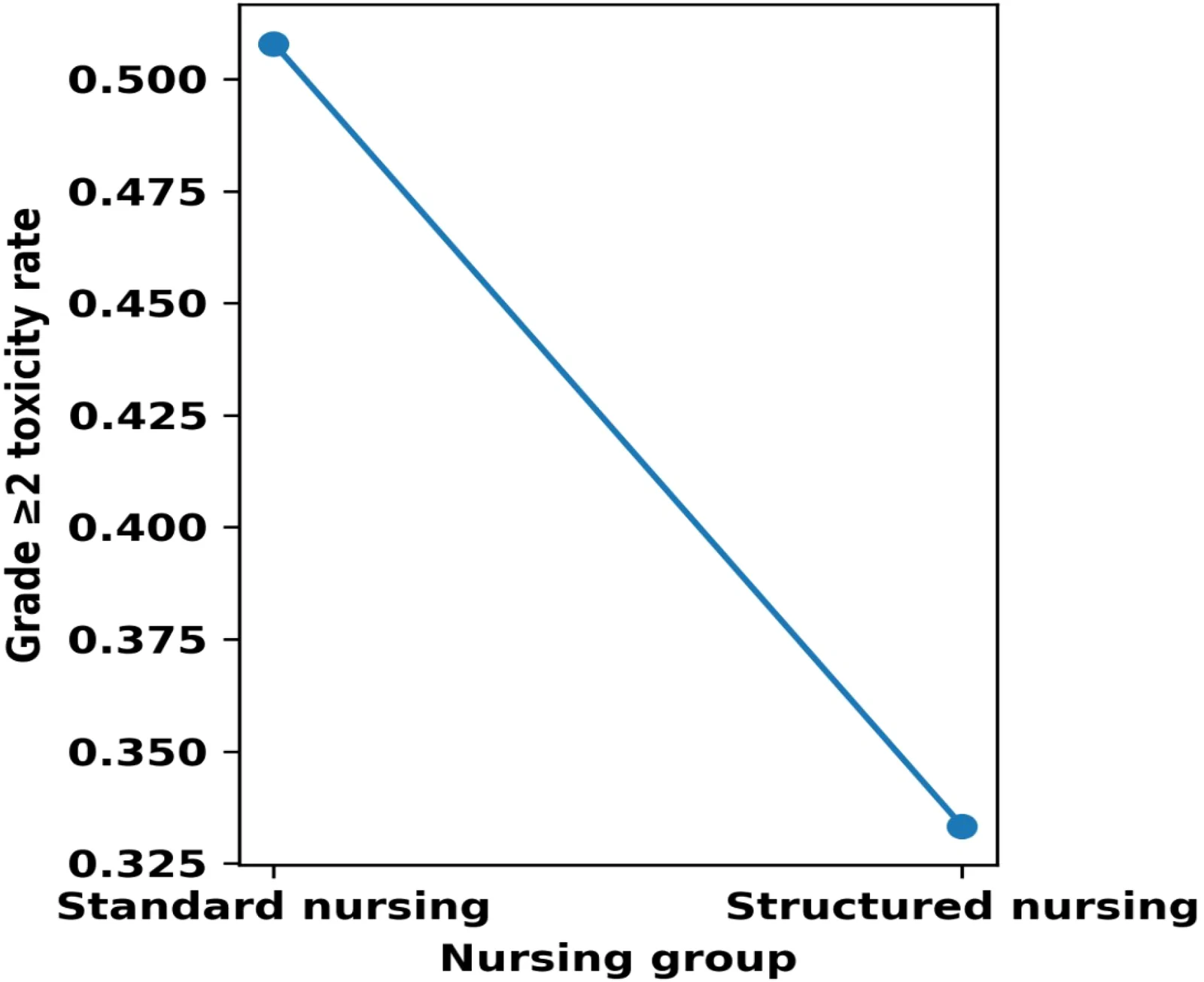
Incidence of Grade ≥2 radiation-related toxicity. Comparison of the incidence of grade ≥2 treatment-related toxicity between structured nursing and standard nursing groups.

**Figure 4:**
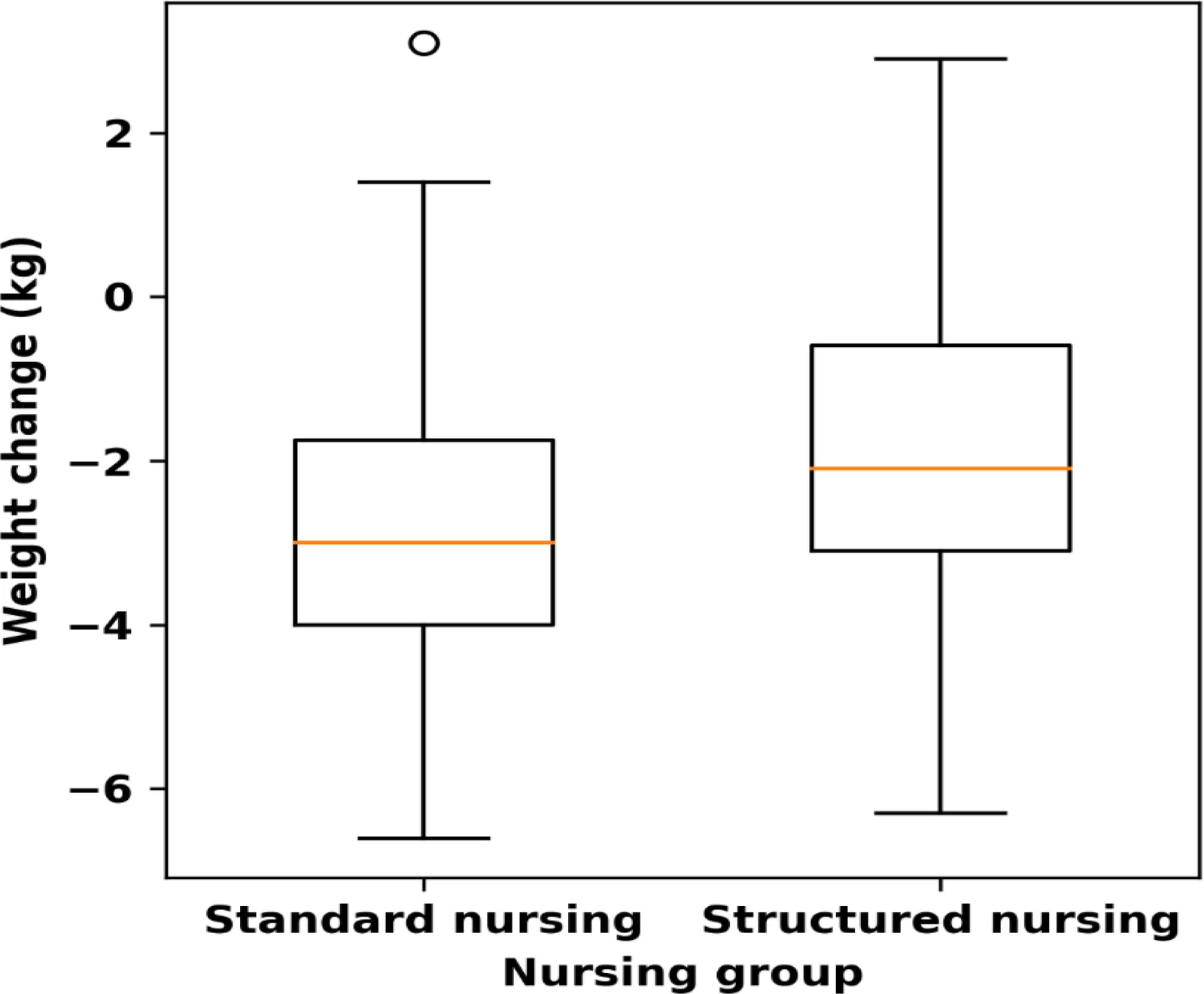
Weight change during radiotherapy by nursing care model. Comparison of body weight change during radiotherapy between structured nursing and standard nursing groups.

Median interruption days were 0 (0–0) in both groups (p = 1.00), indicating that prolonged treatment interruption was uncommon and did not differ between groups.

Overall, these findings demonstrate that structured nursing care was associated with favorable patterns in several treatment tolerance indicators; however, not all observed differences reached statistical significance.

### 3.3 Quality of life outcomes

Quality-of-life outcomes are summarised in **Table 3** and illustrated in **Figure 5**. Global health status, as measured by the EORTC QLQ-C30, declined from baseline to the end of treatment in both groups. However, the decrease was smaller in the structured nursing group (−1.1 ± 6.0) than in the standard nursing group (−2.6 ± 6.1). The QLQ-OES18 symptom score showed a slight decrease (indicating slight improvement) for the structured nursing group (−1.8 ± 6.5), while the symptom score slightly increased for the standard nursing group (+2.3 ± 6.4). Quality-of-life outcomes were assessed descriptively, and changes from baseline to end of treatment were summarized without formal inferential statistical testing. The descriptive results suggest a trend toward greater preservation of overall health status and symptom management in the structured nursing group; however, these data do not permit formal statistical conclusions.

**Table 3:** Quality-of-life outcomes (EORTC QLQ-C30 and QLQ-OES18).

Instrument	Group	Baseline (mean ± SD)	End of RT (mean ± SD)	Change from baseline (mean ± SD)
EORTC QLQ-C30 (0–100; higher=better)	Structured	61.2 ± 9.6	60.1 ± 10.9	-1.1 ± 6.0
EORTC QLQ-C30 (0–100; higher=better)	Standard	64.7 ± 9.4	62.1 ± 11.2	-2.6 ± 6.1
EORTC QLQ-OES18 (0–100; higher=worse)	Structured	53.4 ± 13.8	51.6 ± 16.0	-1.8 ± 6.5
EORTC QLQ-OES18 (0–100; higher=worse)	Standard	50.7 ± 14.0	53.0 ± 15.7	2.3 ± 6.4

Quality of life was measured using the EORTC QLQ-C30 and QLQ-OES18. Scores for both instruments range from 0 to 100. Higher QLQ-C30 scores indicate better global health status, whereas higher QLQ-OES18 scores indicate more severe esophagus-related symptoms. Change scores were calculated by subtracting baseline values from end-of-radiotherapy values.

**Figure 5:**
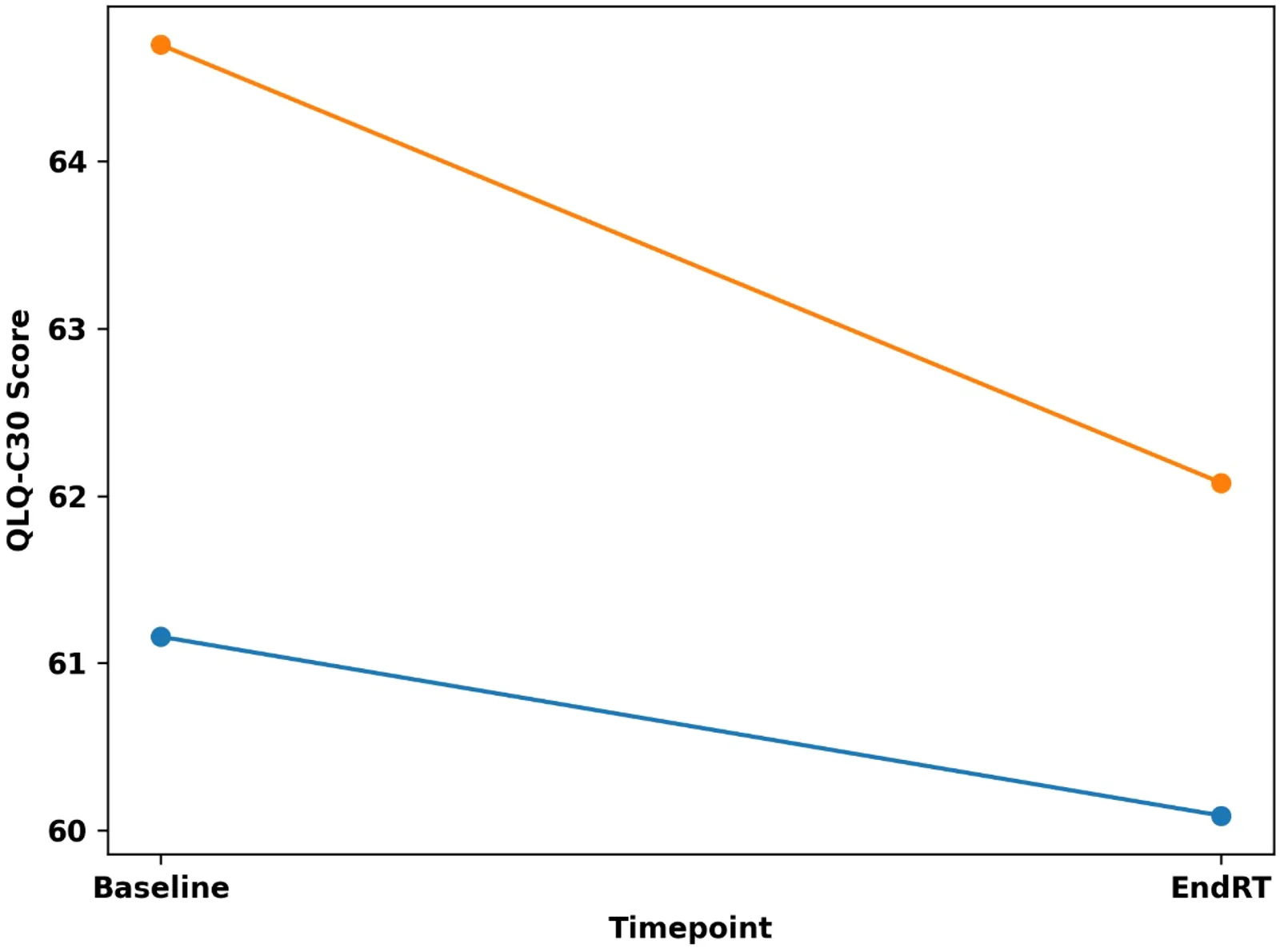
Change in global quality of life (EORTC QLQ-C30) from baseline to end of radiotherapy. Comparison of changes in EORTC QLQ-C30 global health status scores from baseline to end of radiotherapy between structured nursing and standard nursing groups.

### 3.4 Multivariable analysis of radiotherapy completion

Multivariable logistic regression was performed to evaluate the association of several factors with treatment completion (**Table 4**).

**Table 4:** Multivariable logistic regression for radiotherapy completion.

Predictor	OR (95% CI)	P-value
Intercept	3.54 (0.02–592.74)	0.6287
Structured nursing (vs standard)	2.09 (0.69–6.30)	0.1899
Age (per year)	1.03 (0.98–1.08)	0.277
BMI (per 1 kg/m²)	0.95 (0.81–1.11)	0.4965
Stage (per 1 level)	0.92 (0.44–1.91)	0.82
Concurrent chemo (yes vs no)	0.87 (0.32–2.37)	0.7804

Adjusted odds ratios (ORs) with 95% confidence intervals (CIs) were estimated using multivariable logistic regression. The model was adjusted for age, body mass index, tumor stage, concurrent chemotherapy, and the nursing care model.

In the multivariable model, structured nursing care was associated with higher odds of radiotherapy completion, although above 1.0 (i.e., OR 2.09, 95% CI 0.69–6.30), this association was not statistically significant (p = 0.1899).

Age, body mass index, tumor stage, and concurrent chemotherapy were not independently associated with treatment completion in the adjusted model.

Although the direction of effect was consistent with unadjusted findings, the lack of statistical significance indicates that an independent effect of structured nursing could not be confirmed.

These findings should be interpreted as exploratory, given the retrospective observational design and limited sample size.

## 4 Discussion

Esophageal cancer is an extremely serious illness, and the use of radiation therapy is a very important part of its treatment, either to cure or palliate the disease. However, tolerating all aspects of treatment, including radiation, regardless of whether it is curative or palliative, is still very difficult to accomplish due to the effects of radiation-related toxicity, poor nutritional status, and decreased quality of life while receiving treatment. In addition, these factors result in treatment interruptions or incomplete delivery of radiation therapy, leading to worse oncologic outcomes (21–23).

This retrospective cohort study demonstrated that structured nursing care was associated with directionally favorable patterns across a variety of measures of treatment tolerance and quality of life. Compared with patients receiving standard nursing care, patients receiving structured nursing care showed descriptively higher radiotherapy completion rates, a lower incidence of grade 2 or greater toxicity, fewer unplanned hospitalizations, less weight loss, and more favorable descriptive patterns in quality-of-life measures. Although patients in the structured nursing cohort had higher odds of radiotherapy completion in the multivariable model (OR 2.09, 95% CI 0.69–6.30; p = 0.1899), the association was not statistically significant; thus, the findings are exploratory and hypothesis-generating rather than definitive.

Completing planned radiation therapy is an important surrogate endpoint to evaluate how well a patient tolerated their treatment and the quality of care received. Prior research has demonstrated that when patients have their treatments interrupted or terminated early, they have a lower overall survival rate and a higher incidence of local recurrence with their esophageal cancer than patients who complete their planned course of radiation therapy (24–26). In the current study, radiotherapy completion was higher in the structured nursing group, but the analysis adjusted for confounders did not confirm an independent association, suggesting that any observed differences may be attributable to other clinical or treatment-specific characteristics underlying these groups. Although the direction of effect was consistent across analyses, these findings should not be interpreted as evidence of an independent effect but rather as patterns that require confirmation in adequately powered prospective studies (27–29).

The toxicity associated with radiation therapy is one of the most significant impediments to treatment adherence among esophageal cancer patients. The lower rates of ≥grade 2 adverse events and fewer hospitalizations observed in the structured nursing group are consistent with patterns expected under enhanced supportive care delivery. The clinical plausibility of these patterns is supported by structured nursing delivery models that emphasize early symptom identification, timely intervention, and coordinated interdisciplinary care, all of which have been shown in prior studies to improve symptom control (30–32).

Weight loss during the course of radiotherapy is a well-recognized predictor of poor treatment tolerance and adverse outcomes in esophageal cancer (33, 34).

Patients receiving structured nursing care showed less weight loss than those receiving no structured nursing care. This difference may reflect variations in nutritional monitoring and supportive care; however, it cannot be attributed directly to the structured nursing intervention due to potential residual confounding. Prior studies have similarly suggested that enhanced nutritional support and close clinical monitoring are associated with improved treatment tolerance (35, 36).

Quality of life has become an important outcome in the delivery of oncology care today. When using the validated EORTC QLQ-C30 and QLQ-OES18 instruments to assess patients’ quality of life, patients receiving structured nursing demonstrated smaller declines in global health status and more favorable symptom trajectories at a descriptive level.

Nonetheless, longitudinal statistics are not available for comparison; therefore, these should be considered trends rather than statistically significant differences. This evidence is consistent with the existing literature on how organized supportive care interventions positively affect patient-reported outcomes during cancer treatment (37–40).

There are likely many factors working alongside structured nursing care to affect patient outcomes. The mechanisms likely identified thus far include: early identification of adverse events; enhanced treatment adherence; psychosocial support; optimization of nutritional status; and improved coordination of multidisciplinary care for each patient. These mechanisms remain hypothetical within the context of the present study and should not be interpreted as established explanations.

There are several significant limitations to note in this study. First, the retrospective, single-center design limits our ability to draw causal inferences and generalizations. Second, the groups were not randomly assigned to interventions; they were divided based on when the program was initiated and resource availability, which may introduce bias in subject selection. Third, because multivariable adjustment alone is not sufficient to eliminate residual confounding, it remains a possibility. Fourth, there may be confounding prior to treatment initiation due to numerical differences in certain baseline variables, such as body mass index and tumor stage. Fifth, the small sample size reduced our statistical power and resulted in wide confidence intervals, particularly in the multivariable analysis. Sixth, there was no statistically significant association between completion of radiotherapy and structured nursing care after adjustment for covariates in the primary analysis. Seventh, performance status and other potentially important clinical parameters were not included because these data were incomplete. Lastly, long-term oncologic outcome variables, such as survival and disease control, were not evaluated.

Despite these limitations, this study provides clinically relevant observational insights drawn from real-world data, including objective clinical endpoints, standardized toxicity grading, and validated quality-of-life measures. The consistency in direction across multiple outcomes supports the clinical plausibility of these observational patterns; however, confirmation in adequately powered prospective studies is required.

In summary, patients receiving radiotherapy for esophageal cancer with structured nursing support showed directionally more favorable patterns in treatment tolerance and health-related quality-of-life outcomes than those receiving standard nursing care. It should be noted that there was no statistically significant relationship between the provision of structured nursing care and the completion of radiation therapy. Therefore, these results should be interpreted cautiously and should be viewed as exploratory and hypothesis-generating. Further prospective, multicenter studies are needed to determine whether structured nursing interventions improve clinical outcomes.

## 5 Conclusion

In this retrospective single-center cohort of patients receiving radiotherapy for esophageal cancer, structured nursing care was associated with directionally favorable patterns across multiple measures of treatment tolerance and quality of life. However, the adjusted association with radiotherapy completion was not statistically significant (OR 2.09, 95% CI 0.69–6.30; p = 0.1899), and these findings should therefore be interpreted as exploratory rather than confirmatory.

Given the observational design and potential for residual confounding, causal inferences cannot be established. Prospective multicenter studies with standardized supportive-care protocols are needed to determine whether structured nursing care is independently associated with improved treatment tolerance and clinical outcomes in this population.

## Data Availability

The original contributions presented in the study are included in the article/**Supplementary Material**. Further inquiries can be directed to the corresponding author.
