# Supplementary material for: Effect of structured nursing care on treatment tolerance and quality of life in patients with esophageal cancer undergoing radiotherapy: a retrospective cohort study
Source: Front Oncol. 2026 Sep 23;16:1811695. doi: 10.3389/fonc.2026.1811695 (PMC13643691; doi:10.3389/fonc.2026.1811695)
Supplement: Supplementary file 1 [file Table1.docx]

# STROBE Statement – Checklist of Items for Cohort Studies

| Item | Recommendation | Page / Section |
| --- | --- | --- |
| 1a | Study design in title/abstract | Title; Abstract |
| 1b | Informative abstract summary | Abstract |
| 2 | Background/rationale | Introduction |
| 3 | Objectives | End of Introduction |
| 4 | Study design | Methods 2.1 |
| 5 | Setting | Methods 2.1 |
| 6a | Participants eligibility & follow‑up | Methods 2.1 |
| 6b | Matching criteria (if applicable) | Not applicable |
| 7 | Variables | Methods 2.4 |
| 8 | Data sources/measurement | Methods 2.2–2.4 |
| 9 | Bias | Methods 2.1, 2.5; Discussion |
| 10 | Study size | Methods 2.1 |
| 11 | Quantitative variables | Methods 2.5 |
| 12a | Statistical methods | Methods 2.5 |
| 12b | Subgroups/interactions | Not applicable |
| 12c | Missing data | Methods 2.6 |
| 12d | Loss to follow‑up | Not applicable |
| 12e | Sensitivity analyses | Not performed |
| 13a | Participant flow | Results 3.1; Figure 1 |
| 13b | Non‑participation | Results 3.1 |
| 13c | Flow diagram | Figure 1 |
| 14a | Descriptive data | Results Table 1 |
| 14b | Missing data | Methods 2.6 |
| 14c | Follow‑up time | Methods 2.1 |
| 15 | Outcome data | Results Tables 2–3; Figures 2–5 |
| 16a | Main results | Results 3.4; Table 4 |
| 16b | Category boundaries | Methods 2.4 |
| 16c | Absolute risk | Results 3.2 |
| 17 | Other analyses | Results 3.3 |
| 18 | Key results | Discussion |
| 19 | Limitations | Discussion (Limitations) |
| 20 | Interpretation | Discussion |
| 21 | Generalisability | Discussion |
| 22 | Funding | Funding Statement |
